# Association Between the COVID-19 Pandemic and Infant Neurodevelopment: A Comparison Before and During COVID-19

**DOI:** 10.3389/fped.2021.662165

**Published:** 2021-10-06

**Authors:** Peiyuan Huang, Fengjuan Zhou, Yixin Guo, Shanshan Yuan, Shanshan Lin, Jinhua Lu, Si Tu, Minshan Lu, Songying Shen, Antoine Guedeney, Huimin Xia, Xiu Qiu

**Affiliations:** ^1^Division of Birth Cohort Study, Guangzhou Women and Children's Medical Center, Guangzhou Medical University, Guangzhou, China; ^2^Provincial Key Clinical Specialty of Woman and Child Health, Guangdong, China; ^3^Department of Child and Adolescent Psychiatry, Bichat Claude Bernard Hospital, Paris University, Paris, France; ^4^Provincial Clinical Research Center for Child Health, Guangdong, China

**Keywords:** COVID-19 pandemic, child development, infant behavior, social isolation, observational study

## Abstract

**Aim:** To investigate the association between the experience of the coronavirus disease 2019 (COVID-19) pandemic and neurodevelopment of 6-month-old and 1-year-old children and explore the differences in the association by birth order.

**Methods:** This comparison study was embedded in the Born in Guangzhou Cohort Study in China. The exposed group included 546 6-month-old and 285 1-year-old children who attended neurodevelopment assessments between March 1 and May 15, 2020, and the non-exposed group included 3,009 6-month-old and 2,214 1-year-old children during the same months from 2015 to 2019. Neurodevelopment at age 6 months and 1 year was assessed by trained clinical staff using the Ages and Stages Questionnaires, third edition (ASQ-3) and the Gesell Developmental Schedules (GDS).

**Results:** The experience of the pandemic in 2020 was associated with a higher risk of delay in the fine motor (adjusted OR: 2.50, 95% CI: 1.25, 4.99; estimated by logistic regression) and communication (adjusted RR [aRR]: 1.13, 95% CI: 1.02, 1.25; estimated by log-binomial regression) domains at age 1 year. The association between the experience of the pandemic and communication delay at age 1 year only existed in first-born children (aRR: 1.15, 95% CI: 1.03, 1.30) but not in later-born children (aRR: 1.02, 95% CI: 0.84, 1.25). No associations were observed in any domain among 6-month-olds.

**Conclusion:** Experiencing the COVID-19 pandemic and related public health strategies might be associated with a higher risk of delay in the development of fine motor and communication in 1-year-old children; the association observed in the communication domain only existed in first-born children.

## Introduction

The pandemic of COVID-19 (coronavirus disease 2019; the pathogen called SARS-CoV-2, formerly 2019-nCoV) emerged in December 2019 in Wuhan, China (1, 2). As of May 25, 2020, there had been 5,304,772 confirmed cases and 342,029 deaths globally, including 84,536 confirmed cases and 4,645 deaths in China (3). A series of non-pharmaceutical intervention-based public health strategies have been applied in China to control the spread of COVID-19, including isolating confirmed cases, contact tracing, quarantine of exposed persons, travel restrictions, school and workplace closures, cancellation of mass gatherings, etc. (4). While such strategies have effectively controlled COVID-19 transmission across China and substantially reduced the number of cases (4), it has been suggested that these strategies may be associated with poorer mental health and development of school-aged children and adolescents (5–7). However, whether this association exists among children at a younger age (e.g., infants) has not been investigated.

A range of biological and psychosocial factors, such as maternal physical and mental health, physical activity, socioeconomic status, and family context, are associated with child neurodevelopment (8, 9). During the COVID-19 pandemic, the implementation of containment strategies reduces children's outdoor activities and their opportunities to contact people other than their family members. Staying indoor may also increase the screen time of the children and their family members (10). Moreover, the pandemic has led to increased anxiety and depression in the population (11), and mental health issues of parents and caregivers may have negative impacts on child development (12).

It is also suggested that neurodevelopment between first-born and later-born children may be different (13). First-born and later-born children may react differently during the COVID-19 pandemic. As two-child families have become increasingly common in China due to the relaxation of the one-child policy, it is worth studying the association between the experience of the COVID-19 pandemic and the neurodevelopment in first-born and second-born children.

As a megacity with a population of over 15,000,000 (14), Guangzhou has also been influenced by the COVID-19 pandemic. Following the lockdown of Wuhan and other cities in Hubei province from January 23, residents in Guangzhou had been encouraged to reduce outdoor activities and maintain social distancing. As mentioned above, the experience of public health strategies in response to COVID-19 might potentially influence child neurodevelopment in several ways. Investigating the association of this experience with infant neurodevelopment can help comprehensively evaluate the influence of COVID-19 and the associated strategies on the population and inform public health policies for both infectious disease control and child health in the future. The aim of the present study was to investigate the association between the experience of the COVID-19 pandemic and neurodevelopment of children aged 6 months and 1 year and explore the differences in the association by birth order.

## Methods

### Study Population

This comparison analysis was part of the Born in Guangzhou Cohort Study (BIGCS), an ongoing prospective birth cohort conducted in the Guangzhou Women and Children's Medical Center (GWCMC), China. Details of the BIGCS have been described elsewhere (15). In brief, women were recruited during their first routine antenatal examinations in early pregnancy (<20 weeks of gestation, normally at around 16 weeks) at two campuses of the GWCMC, and followed up in mid- (at 24–28 weeks) and late pregnancy (at 35–38 weeks). After delivery, the women and their children were followed up at 6 weeks, 6, 12, 24, and 36 months postpartum. The protocol of the BIGCS was approved by the Institutional Ethics Committee of the GWCMC. All participants provided written informed consent at the time of recruitment.

**Figure 1:**
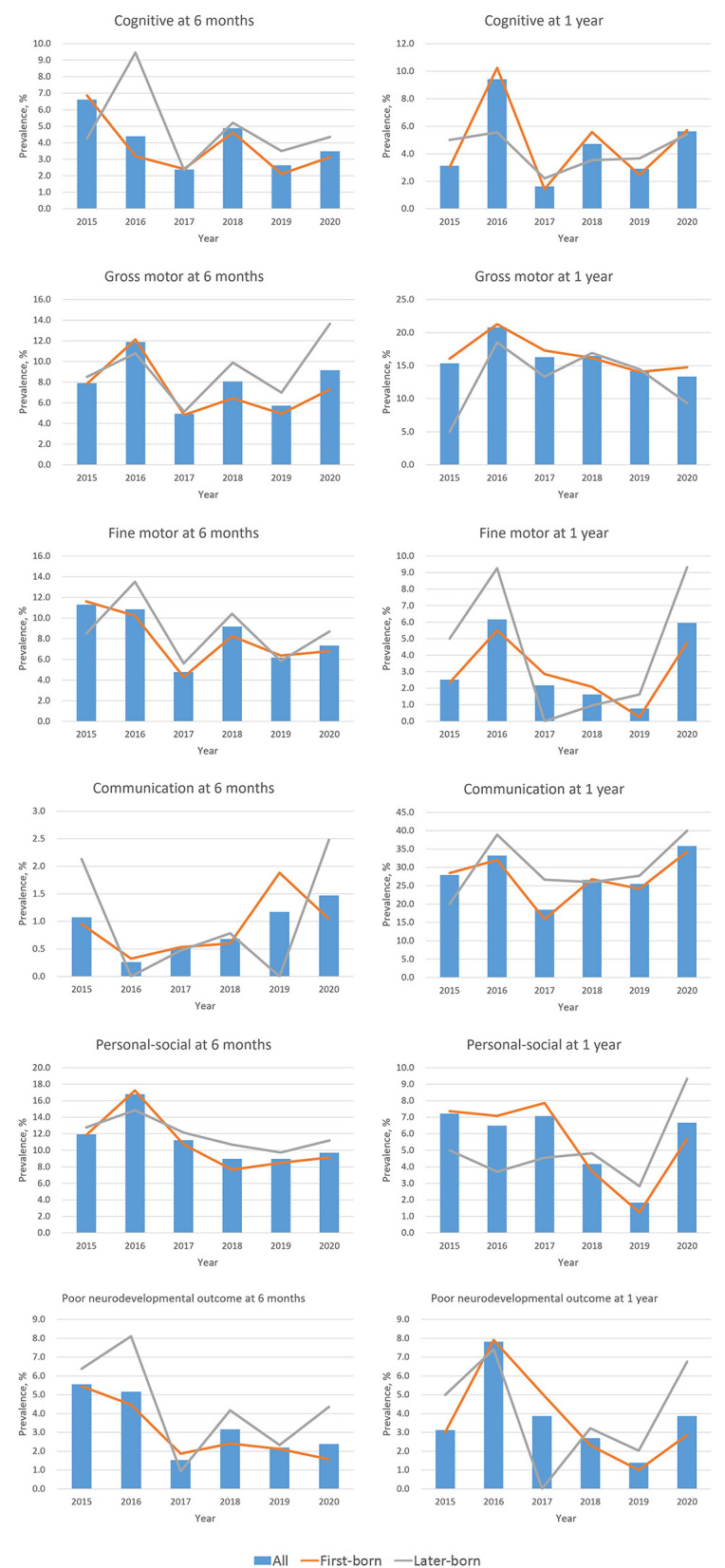
Secular trend for the prevalence of neurodevelopmental delay at age 6 months and 1 year. Neurodevelopment was measured by the Ages & Stages Questionnaires, 3rd Edition (ASQ-3) at age 6 months, and by the Gesell Developmental Schedule (GDS) at age 1 year. Poor neurodevelopmental outcome was defined as developmental delay in more than two domains.

The routine follow-up of the BIGCS had been suspended since the outbreak of COVID-19 in late January and was resumed on March 1, 2020. The present study included the children who attended the neurodevelopmental assessment at the 6-month or 1-year follow-up during March 1–May 15, 2020, as the exposed group, and those during the same months from 2015 to 2019 as the non-exposed group. Children were excluded if they withdrew before the present study, had multiple births, were preterm births (defined as gestational weeks at birth <37), had birth hypoxia (defined as Apgar score ≤ 7 at either 1 or 5 min), or had birth defects that might affect neurodevelopmental outcomes (including major birth defects and anomalies that affect language and motor functions). The flowchart for the selection process of the present study is shown in Figure 1.

### Assessment of Neurodevelopment

Children's neurodevelopment at the 6-month and 1-year follow-up was assessed by clinical staff using the Ages and Stages Questionnaires, third edition (ASQ-3) and the Gesell Developmental Schedules (GDS), respectively. Before the field work, all clinical evaluators had attended training courses accredited by the official providers of these assessment tools. All assessments were conducted strictly following the instruction manuals. Regular training and quality control sessions were held by the BIGCS team to perform live examinations for each evaluator, monitor any potential assessment errors, and review all the assessment results to ensure internal consistency and data quality.

### GDS

The GDS was used to evaluate the developmental quotient (DQ) in children's adaptive (i.e., cognitive), gross motor, fine motor, language (i.e., communication), and personal-social domains at the 1-year follow-up (16). The Chinese version of the GDS has been validated and adopted by the Chinese Pediatric Association (17, 18). The sum of adaptive, language and personal-social DQ was significantly correlated to the mental development index of Bayley Scales of Infant Development (BSID, *r* = 0.75, *P* < 0.0001), and the sum of gross motor and fine motor DQ was significantly correlated to the psychomotor development index of BSID (*r* = 0.55, *P* < 0.05) (19, 20). Higher DQ reflects better neurodevelopmental performance. As with other child development studies using the Chinese version of GDS, we defined developmental delay in each domain as DQ <86 (21–23). To use a composite indicator to reflect the severity of adverse developmental outcomes across all five domains in the present study, we defined “poor neurodevelopmental outcome” as developmental delay in more than two domains on the GDS.

### ASQ-3

The ASQ-3 is a comprehensive standardized developmental monitoring tool for children from 1 to 66 months of age. At the 6-month follow-up, the ASQ-3 was administered by trained psychological evaluators at the GWCMC's child health care clinics. Similar to the GDS, it also assesses the following five developmental domains: communication, gross motor, fine motor, problem-solving (i.e., cognitive), and personal-social, with the cutoffs for developmental delay varying across different domains and ages (24, 25). The ASQ-3 has generally high internal consistency, test-rest reliability, and acceptable sensitivity in the Chinese population (26). To be consistent with the results from the GDS, “poor neurodevelopmental outcome” on the ASQ-3 was also defined as developmental delay in more than two domains.

### Other Variables

Information on a range of maternal and child characteristics was collected from questionnaires or medical records from early pregnancy to age 1 year of the children. Maternal characteristics included age at conception (in years), monthly income (<1,500, 1,500–4,500, 4,501–9,000, or ≥9,001 yuan, based on the individual income tax brackets implemented since January 1, 2012), education level (high school or below, college, undergraduate, or postgraduate), tobacco exposure in early pregnancy (yes/no), pre-pregnancy body mass index (BMI, calculated as pre-pregnancy weight in kilogram divided by squared height in meter), gestational diabetes mellitus (GDM, yes/no), hypertensive disorders of pregnancy (HDP, yes/no), depressive symptoms in early pregnancy (Self-rating Depression Scale score ≥53) (27, 28), anxiety symptoms in early pregnancy (Self-rating Anxiety Scale score ≥50) (29, 30), and depressive symptoms at 6 months or 1 year postpartum (Edinburgh Postnatal Depression Scale score ≥13) (31). Child characteristics at birth included gestational age at birth (in weeks), child sex (male/female), birth order (1 or >1), birth weight z-score (calculated based on the INTERGROWTH-21st standards) (32), delivery mode (vaginal birth or cesarean section). Child characteristics at age 6 months or 1 year included child age (in months), duration of breastfeeding (in days), number of other children living together (0 or >0), and anthropometric indicators including BMI (calculated as children's weight in kilogram divided by squared height in meter) and head circumference, both calculated as z-scores based on the standards from the World Health Organization (33).

### Statistical Analyses

Descriptive statistics, i.e., mean (standard deviation) and frequencies (percentages), were reported for all maternal and child characteristics. For both ages, the prevalence of delay in each domain and “poor neurodevelopmental outcome” was presented by year. χ^2^-test was used to compare the prevalence of developmental delay in different years.

The associations between the experience of the COVID-19 pandemic (i.e., the year 2020 vs. 2015–2019) and the risk of neurodevelopmental delay at age 6 months and 1 year were examined using log-binomial regression (for the outcomes with a prevalence ≥15%, e.g., developmental delay in the gross motor and the communication domains at age 1 year, shown as relative risk [RR]) and logistic regression (for all other outcomes, shown as odds ratio [OR]), respectively. The models were adjusted for maternal age, education level, monthly income, delivery mode, child sex, birth order, birth weight z-score, breastfeeding duration, BMI z-score, head circumference z-score, and maternal postpartum depressive symptoms. Selection of the covariates is based on the following considerations: firstly, we took into account maternal demographic and socioeconomic factors (represented by maternal age, education level, and monthly income), which have been shown to be associated with child development; (34, 35) secondly, children's developmental outcomes might also differ by some factors at delivery, such as delivery mode, child sex, and birth order; (13, 36, 37) thirdly, birth weight z-score, breastfeeding duration, BMI z-score, and head circumference z-score generally reflect the child's intrauterine and postnatal growth and nutritional status, which are important factors related to neurodevelopment; (38) lastly, maternal postpartum mental illness (e.g., postpartum depression) was also considered, as evidence has suggested that it might have negative impacts on infants' developmental outcomes (39). Moreover, stratification analyses by birth order were also performed to explore the association between the experience of the COVID-19 pandemic and neurodevelopmental outcomes in first-born and later-born children.

Two-sided *P* < 0.05 was considered statistically significant. All analyses were performed using SAS 9.3 software (SAS Institute, Cary, NC, USA).

## Results

The flowchart for selecting the study population is shown in Supplementary Figure 1. Between March 1 and May 15 from 2015 to 2020, a total of 3,937 children in the BIGCS attended neurodevelopment assessments at around 6 months of age. After excluding those who withdrew before the present study (*N* = 10), were multiple births (*N* = 147), were preterm births (*N* = 182), had hypoxia at birth (*N* = 18), had neurodevelopment-related birth defects (*N* = 25), there were 3,555 children included in the analysis for the 6-month follow-up. During the same period, a total of 2,777 children in the BIGCS attended neurodevelopment assessments at around 1 year of age. We excluded those who withdrew before the present study (*N* = 5), were multiple births (*N* = 105), were preterm births (*N* = 121), had hypoxia at birth (*N* = 15), had neurodevelopment-related birth defects (*N* = 32), resulting in 2,499 children included in the analysis for the 1-year follow-up. Among those who remained in the analysis, 546 6-month-old and 285 1-year-old children who attended the neurodevelopment assessment between March 1 and May 15, 2020, were included as the exposed group, while 3,009 6-month-old and 2,214 1-year-old children during the same months from 2015 to 2019 were included as the non-exposed group.

Maternal and child characteristics are shown in Table 1, with data presented separately between the follow-ups at 6 months and 1 year, and between 2015–2019 and 2020. Maternal age at conception was around 30 years. As for child characteristics, gestational age at birth was around 39 weeks, and the proportion of boys was 52–54%. In 2020, the proportion of children born via cesarean section was lower than that in 201–2019. At the time of neurodevelopment assessment, the mean age of children included in the 6-month and 1-year follow-up was 6.24 and 12.31 months, respectively.

**Table 1:** Maternal and child characteristics by year.

	**Age 6 months**				**Age 1 year**		
	**Total (*N =* 3,555)**	**2015–2019 (*N =* 3,009)**	**2020 (*N =* 546)**	**P**	**Total (*N =* 2,499)**	**2015–2019 (*N =* 2,214)**	**2020 (*N =* 285)**	**P**
**Maternal characteristics**								
Age at conception (years), mean (SD)	30.3 (3.9)	30.3 (3.9)	30.1 (3.8)	0.414	30.2 (3.8)	30.2 (3.8)	30.2 (3.6)	0.89
**Monthly income (yuan)**, ***n (%)***				<0.001				<0.001
≤ 1,500	307 (9.0)	274 (9.3)	33 (6.0)		209 (8.4)	193 (8.8)	16 (5.6)	
1,501–4,500	631 (18.5)	584 (19.8)	47 (8.6)		502 (20.3)	465 (21.2)	37 (13.1)	
4,501–9,000	1,405 (41.2)	1,225 (41.5)	180 (33.0)		1,043 (42.2)	923 (42.1)	120 (42.4)	
≥9,001	927 (27.2)	747 (25.3)	180 (33.0)		628 (25.4)	534 (24.4)	94 (33.2)	
Refused to answer	143 (4.2)	118 (4.0)	106 (19.4)		92 (3.7)	76 (3.5)	16 (5.6)	
**Education level**, ***n (%)***				<0.001				0.029
High school or below	286 (8.0)	254 (8.4)	32 (5.9)		185 (7.4)	173 (7.8)	12 (4.2)	
College	763 (21.5)	651 (21.6)	112 (20.5)		545 (21.8)	497 (22.4)	48 (16.8)	
Undergraduate	2,025 (57.0)	1,709 (56.8)	316 (57.9)		1,405 (56.2)	1,220 (55.1)	185 (64.9)	
Postgraduate	481 (13.5)	395 (13.1)	86 (15.7)		364 (14.6)	324 (14.6)	40 (14.0)	
Tobacco exposure in early pregnancy, *n (%)*	936 (27.4)	817 (27.7)	119 (25.6)	0.311	734 (29.7)	664 (30.3)	70 (24.9)	0.043
**Pre-pregnancy body mass index (kg/m**^**2**^**)**, ***n (%)***				0.369				0.707
<18.5	697 (21.2)	601 (21.1)	96 (21.5)		507 (20.9)	456 (21.3)	51 (18.3)	
18.5–23.9	2,220 (67.4)	1,917 (67.3)	303 (67.9)		1,645 (68.0)	1,447 (67.6)	198 (71.0)	
≥24.0	376 (11.4)	329 (11.6)	47 (10.5)		268 (11.1)	238 (11.1)	30 (10.8)	
Gestational diabetes mellitus, *n (%)*	541 (15.4)	457 (15.4)	84 (15.5)	0.949	382 (15.6)	336 (15.5)	46 (16.3)	0.707
Hypertensive disorders of pregnancy, *n (%)*	114 (3.2)	88 (2.8)	32 (5.9)	<0.001	86 (3.5)	80 (3.7)	6 (2.1)	0.178
With depressive symptoms in early pregnancy, *n (%)*	686 (20.5)	581 (20. 1)	105 (22.9)	0.148	527 (21.7)	463 (21.6)	64 (22.8)	0.632
With anxiety symptoms in early pregnancy, *n (%)*	446 (13.3)	388 (13.4)	58 (12.6)	0.645	348 (14.3)	301 (14.0)	47 (16.7)	0.202
With postpartum depressive symptoms at the time of child neurodevelopment assessment, *n (%)*	599 (17.8)	507 (17.6)	92 (19.1)	0.398	380 (16.0)	321 (15.2)	59 (22.6)	0.001
**Child characteristics (at birth)**								
Gestational age at birth (weeks), mean (SD)	39.0 (1.0)	39.0 (1.0)	39.1 (1.0)	0.146	39.0 (1.0)	39.0 (1.0)	39.0 (1.0)	0.405
Boys, *n (%)*	1,888 (53.1)	1,594 (53.0)	294 (53.9)	0.631	1,320 (52.9)	1,165 (52.7)	155 (54.4)	0.522
Birth order >1, *n (%)*	1,137 (32.0)	976 (32.4)	161 (29.6)	0.155	756 (30.3)	681 (30.8)	75 (26.3)	0.098
Birth weight z-score, mean (SD)	−0.1 (0.8)	−0.1 (0.8)	0.0 (0.8)	0.03	0.0 (0.8)	0.0 (0.8)	0.1 (0.8)	0.129
Cesarean section, *n (%)*	1,043 (29.5)	907 (30.3)	136 (25.0)	0.002	776 (31.3)	710 (32.4)	66 (23.2)	<0.001
**Child characteristics (at the time of neurodevelopment assessment)**								
Age (months), mean (SD)	6.2 (0.5)	6.2 (0.4)	6.4 (0.6)	<0.001	12.3 (0.6)	12.3 (0.6)	12.6 (0.7)	<0.001
Duration of breast feeding (days), mean (SD)	169.6 (73.3)	167.9 (76.6)	180.7 (43.2)	<0.001	279.6 (104.2)	278.7 (104.0)	287.3 (106.0)	0.233
Body mass index z-score, mean (SD)	0.2 (1.0)	0.2 (1.0)	0.2 (1.0)	0.769	0.1 (0.9)	0.1 (0.9)	0.0 (0.9)	0.204
Head circumference z-score, mean (SD)	0.2 (0.9)	0.2 (0.9)	0.3 (0.9)	0.223	0.0 (0.9)	0.0 (1.0)	0.0 (0.9)	0.705

*Percentages may not add up to 100% due to rounding*.

*P-values represent the statistical significance for the difference between 2015–2019 and 2020, tested by the t-test (for continuous variables) and χ^2^ test (for categoricalvariables)*.

*SD, standarddeviation*.

**Figure 2:**
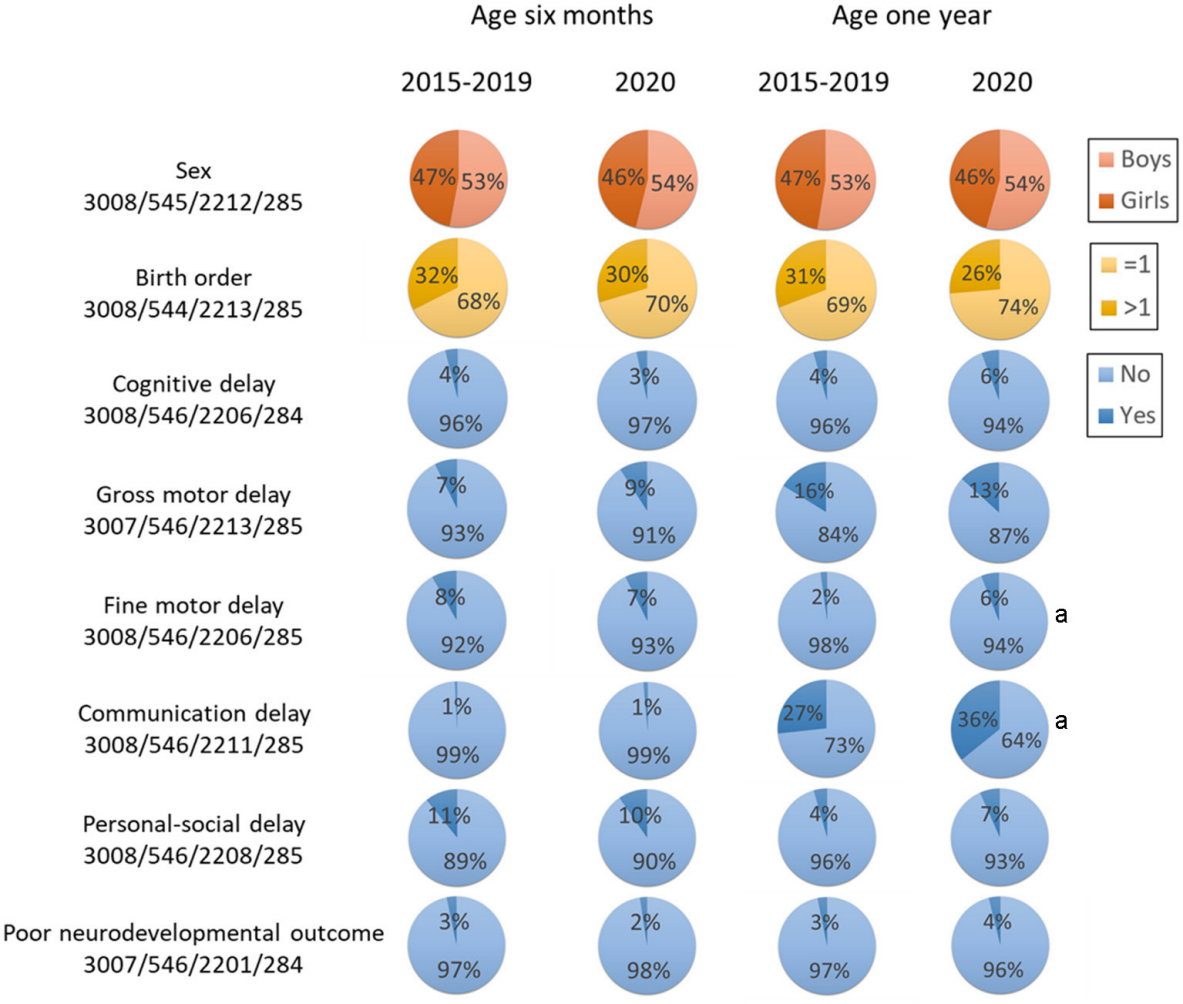
Key characteristics by age and year. Poor neurodevelopmental outcome was defined as developmental delay in more than two domains. ^a^Statistical significance compared with 2020 at the same age.

Figure 1 shows the age- and domain-specific trend of neurodevelopmental delay from 2015 to 2020 (see detailed data in Supplementary Table 1), whereas Figure 2 shows the proportion of child sex, birth order, and neurodevelopmental delay in each domain by age in 2015–2019 and 2020, respectively. In 6-month-old children, the prevalence of neurodevelopmental delay assessed by the ASQ-3 fluctuated over the 6 years across all domains; and there were no differences in these proportions between 2015–2019 and 2020. At 1 year of age, while fluctuations in the prevalence of neurodevelopmental delay assessed by the GDS were also observed, the children in 2020 had a higher proportion of delay in the fine motor and the communication domains (both *P* < 0.05) than those in the previous years. Differences in the prevalence of neurodevelopmental delay were also observed between first-born and later-born children (Figure 1).

The association between the experience of COVID-19 and the risk of neurodevelopmental delay are shown in Table 2. No associations were observed in any domain at 6 months of age. Experiencing COVID-19 was associated with a higher risk of delay in the fine motor (adjusted OR [aOR]: 2.50, 95% CI: 1.25, 4.99) and communication (adjusted RR [aRR]: 1.13, 95% CI: 1.02, 1.25) domains at 1 year of age. Table 3 shows the associations between the experience of COVID-19 and the risk of neurodevelopmental delay, stratified by birth order. At age 1 year, experiencing the COVID-19 pandemic in 2020 was associated with a higher risk of communication delay (aRR: 1.15, 95% CI: 1.13, 1.30) in first-born children, while this association was not observed in those who were later-born (aRR: 1.02, 95% CI: 0.84, 1.25). Notably, no associations with “poor neurodevelopmental outcome” were found in all analyses above.

**Table 2:** Association between the experience of COVID-19 and child neurodevelopment at age 6 months and 1 year in all samples.

**2020 vs. 2015–2019**	**Age 6 months**		**Age 1 year**	
	**Crude OR (95% CI)**	**Adjusted OR (95% CI)^a^**	**Crude OR or RR (95% CI)**	**Adjusted OR or RR (95% CI)^a^**
Cognitive delay	0.85 (0.52, 1.38)	0.84 (0.46, 1.56)	1.31 (0.76, 2.26)	1.12 (0.58, 2.14)
Gross motor delay	1.26 (0.92, 1.74)	1.30 (0.89, 1.89)	0.82 (0.60, 1.12)^b^	0.91 (0.76, 1.09)^b^
Fine motor delay	0.89 (0.63, 1.26)	0.97 (0.65, 1.45)	2.85 (1.62, 5.03)^*^	2.50 (1.25, 4.99)^*^
Communication delay	1.93 (0.86, 4.34)	2.09 (0.87, 5.01)	1.34 (1.13, 1.59)^b^^*^	1.13 (1.02, 1.25)^b^^*^
Personal-social delay	0.88 (0.65, 1.20)	0.82 (0.56, 1.20)	1.52 (0.92, 2.53)	1.12 (0.56, 2.22)
Poor neurodevelopmental outcome^c^	0.72 (0.40, 1.30)	0.90 (0.46, 1.77)	1.23 (0.64, 2.35)	0.77 (0.30, 1.96)

* *P <0.05*.

a *Adjusted for maternal age, education level, monthly income, delivery mode, child sex, birth order, birth weight z-score, breastfeeding duration, body mass index z-score, head circumference z-score, and maternal postpartum depressive symptoms*.

b *Estimated by log-binomial regression, shown as RR*.

c *Defined as developmental delay in more than two domains*.

*CI, confidence interval; OR, odds ratio; RR, relative risk*.

**Table 3:** Association between the experience of COVID-19 and child neurodevelopment at age 6 months and 1 year, stratified by birth order.

**2020 vs. 2015–2019**	**Age 6 months**		**Age 1 year**	
	**First-born**	**Later-born**	**First-born**	**Later-born**
Cognitive delay—Adjusted OR (95% CI)^a^	0.90 (0.44, 1.85)	0.73 (0.22, 2.44)	1.09 (0.51, 2.34)	1.20 (0.32, 4.45)
Gross motor delay—Adjusted OR or RR (95% CI)^a^	1.12 (0.69, 1.81)	1.73 (0.92, 3.24)	0.98 (0.81, 1.18)^b^	0.69 (0.43, 1.13)^b^
Fine motor delay—Adjusted OR (95% CI)^a^	0.96 (0.59, 1.56)	1.05 (0.50, 2.21)	2.07 (0.88, 4.86)	3.47 (0.94, 12.90)
Communication delay—Adjusted OR or RR (95% CI)^a^	1.56 (0.51, 4.79)	**–** ^c^	1.15 (1.03, 1.30)^b^^*^	1.02 (0.84, 1.25)^b^
Personal-social delay—Adjusted OR (95% CI)^a^	0.80 (0.51, 1.26)	0.83 (0.41, 1.66)	0.71 (0.28, 1.83)	2.74 (0.92, 8.13)
Poor neurodevelopment outcome^d^–Adjusted OR (95% CI)^a^	0.64 (0.25, 1.64)	1.64 (0.60, 4.44)	0.41 (0.10, 1.75)	1.87 (0.48, 7.21)

* *P <0.05*.

a *Adjusted for maternal age, education level, monthly income, delivery mode, child sex, birth weight z-score, breastfeeding duration, body mass index z-score, head circumference z-score, and maternal postpartum depressive symptoms*.

b *Estimated by log-binomial regression, shown as RR*.

c *Omitted due to the small sample size*.

d *Defined as developmental delay in more than two domains*.

*CI, confidence interval; OR, odds ratio; RR, relative risk*.

## Discussion

To our knowledge, this is the first study to reveal the risk of neurodevelopmental delay in infants during the COVID-19 pandemic. In this comparison study with information on infant neurodevelopment from 2015 to 2020, we found that experiencing COVID-19 in 2020 was associated with a higher risk of neurodevelopmental delay in the fine motor and the communication domains in 1-year-old children, while no associations are observed for those at 6 months of age. The delay in the communication domain at age 1 year is mainly attributable to first-born children. Experiencing the pandemic was not associated with “poor neurodevelopmental outcome” at either age.

Although there are currently no other studies specifically investigating the influence of the COVID-19 pandemic and related prevention and control strategies on infant neurodevelopment, previous studies have shown that the COVID-19 pandemic and associated strategies (e.g., social isolation) may have a negative impact on the development and mental health of preschool-age and school-age children and adolescents. For example, a systematic review shows that children and adolescents are more likely to experience depression and anxiety during and after enforced social isolation (6). Similarly, other studies also reveal the reduction of life satisfaction and the exacerbation of psychiatric disorders in adolescents during the pandemic (5, 7). Our study adds new evidence to the literature regarding the association between the experience of the COVID-19 pandemic and neurodevelopment of infants. For the first time, our findings suggest that experiencing the COVID-19 pandemic may be associated with compromised neurodevelopment at 1 year of age, a critical window for the development of specific domain functions (40).

We observed that 1-year-old children experiencing the pandemic were at an increased risk of delay in the communication (language) and the fine motor domains than those not. Evidence shows that the protective factors of 1-year-old children who are in “at-risk” environments for language development include parental self-efficacy, relationship well-being, high social support, community participation, and daily parent-child interaction (41). The COVID-19 pandemic might have negatively affected the mental health of the children's parents and reduced the contact with other family members, friends, and community members due to the containment strategies, thus potentially exerting a negative impact on children's language development. Fine motor skills are hand and finger skills such as picking up pellets with their thumb and forefinger. When staying indoors during the pandemic, some parents and caregivers (e.g., grandparents) might entertain their children with digital devices, thus increasing their screen time. Evidence (although from children of preschool age) has suggested that those who extensively use touch screen tablets might have poorer fine motor development than those who do not (42).

Our study also found that the association between the experience of COVID-19 and the risk of neurodevelopmental delay in specific domains among 1-year-olds only existed in first-born children, which seems inconsistent with previous evidence. It has been shown that first-born children tend to be more intelligent and receive more language interactions from their parents than later-born children (43, 44). There might be two reasons for this. First, during the pandemic, the children stayed at home all day with their elder brothers or sisters, and the extensive interactions with their elder siblings might alleviate the potentially negative impact of social distancing and home confinement measures. Second, as the pandemic has universally caused anxiety and depression in the general population (45), new parents may be less experienced in coping with these emotions, and the infants born to them might be more vulnerable to developmental issues. However, these findings still need to be replicated and confirmed by other studies in the future.

The different findings between 6-month-olds and 1-year-olds in our study are also noteworthy, which might be attributed to the differences in the sensitive periods for neurodevelopment and the assessment tools used in the two groups. For the children included in the present study, most of them experienced the COVID-19 pandemic at age 4–6 or 10–12 months. Evidence has shown that children reared institutionally at 4–6 months of age did not have a significantly increased risk of adverse developmental outcomes across most domains compared with their non-institutionalized counterparts, (46) suggesting that this period might be less sensitive to negative impacts, which is in line with our findings for 6-month-olds. On the other hand, infants start to discriminate native and non-native phonemes at age 6–12 months (47), rendering this period a likely sensitive time window for language/communication development, which is also supported by our findings for 1-year-olds. Although there is a lack of evidence on the sensitive periods for fine motor development, especially within infancy, a study of preschool-aged children has found some sensitive periods for fine motor development during 2.8–6.5 years of age (48). Our findings also suggest the possible existence of sensitive periods for fine motor development within infancy, which is worth further exploration. In addition, the different assessment scales used in these two groups could be an alternate explanation. Unlike the GDS, which is a diagnostic tool and often used as a reference in validation studies, the ASQ is a concise screening tool suitable for use in large-scale epidemiologic studies. As the GDS contains more items and has a more detailed assessment and scoring process than the ASQ, we cannot rule out the possibility that the ASQ might have failed to identify any subtle differences in the development of 6-month-olds. Therefore, the results of these two groups might not be directly comparable with each other and should be interpreted independently.

The present study has several strengths. Firstly, this is the first study to examine the relationship between the experience of the COVID-19 pandemic and infant neurodevelopment. Secondly, information on the variables used in this study was prospectively collected, and the analyses were adjusted for a range of potential confounders. Thirdly, children's neurodevelopment at 6 months and 1 year old was examined by trained clinical staff.

Our study also has some limitations. Firstly, we could not obtain the information on the degree to which the children's families complied with the social distancing and home confinement strategies. It is likely that not all families kept social distancing and stayed at home during the pandemic, and those who less cared about the strategies seemed more likely to attend the 6-month or 1-year follow-ups in the hospital. Secondly, although we stratified the children by birth order, the number of family members living together with the children is unknown, which might also influence child neurodevelopment. Thirdly, due to the different assessment tools used in the 6-months and 1-year children, the results might not be directly comparable between these two age groups. Fourthly, we could not rule out the possible bias caused by the fluctuation of outcome prevalence over time. To alleviate this issue, we combined the data from 2015 to 2019 in the analysis to obtain a relatively stable outcome prevalence for the reference group. In addition, this study only included neurodevelopment within infancy, and longer follow-ups are thus needed to explore the potential influence of this experience on long-term developmental outcomes of children. Moreover, this study was based on the Chinese population, which limits the generalizability of our findings. Replication by future studies from other regions and populations is needed. Lastly, residue confounding might exist due to the observational nature of the present study.

Several implications can be drawn from this study. First, our findings suggest that the experience of the COVID-19 pandemic might potentially have a negative impact on child neurodevelopment in specific domains at specific ages, which raise concerns about the development of young children under the COVID-19 pandemic. Parents need to pay more attention to their children's development and adjust their rearing patterns at this special time (44). Second, given the absence of an association with “poor neurodevelopmental outcome,” this potential negative impact seemed to affect only a limited number of domains and have minor influences on the overall developmental outcome of the children. Third, for families with two or more children, the key to minimize the impact of the pandemic on the children's development might be to increase the interaction of children with their siblings and other family members. Fourth, for child healthcare practitioners, it is necessary to consider using the internet, such as social media and other communication software, to practice health education and provide online assessment and intervention instructions for parents during the pandemic (49, 50). Moreover, studies are needed to further explore the impact of COVID-19 on child health and its underlying mechanisms. Evidence has suggested that pregnant women tend to report adverse lifestyle changes during the COVID-19 pandemic (e.g., having poorer diet quality and less sleep, and being less physically active); (51) some of these changes might, in turn, influence offspring's physical and mental health, such as neurodevelopment (52, 53). Thus, future research is warranted to investigate the potential influence of COVID-19 on offspring health mediated by the changes in women's behaviors and lifestyles during pregnancy.

In conclusion, this study found an association between the experience of COVID-19 and a higher risk of neurodevelopmental delay in the fine motor and the communication domains in 1-year-old children; the association observed in the communication domain only existed in first-born children. No associations were found in 6-month-old children. These findings underline the need to concern about the neurodevelopment of infants who are experiencing the COVID-19 pandemic and call for more training in specific domains at home, which requires the joint efforts of both parents and child healthcare practitioners.

## Data Availability Statement

The original contributions presented in the study are included in the article/Supplementary Material, further inquiries can be directed to the corresponding author/s.

## Ethics Statement

The studies involving human participants were reviewed and approved by The Institutional Ethics Committee of Guangzhou Women and Children's Medical Center. Written informed consent to participate in this study was provided by the participants' legal guardian/next of kin.

## Author Contributions

HX and XQ conceptualized and designed the study, supervised its implementation, and reviewed and revised the manuscript. PH and FZ carried out data analysis, drafted the initial manuscript, and reviewed and revised the manuscript. YG, SY, SL, JL, ST, ML, and SS were involved in study design, questionnaire development, data collection and management, follow-up of participants, and critically reviewed the manuscript for important intellectual content. AG interpreted the data and critically reviewed the manuscript for important intellectual content. All authors approved the final manuscript as submitted and agree to be accountable for all aspects of the work.

## Funding

This study was supported by National Natural Science Foundation of China (31900608), Department of Science and Technology of Guangdong Province, China (2019B030316014), and Guangzhou Municipal Health Commission (20191A031002 and 20161A011023).

## Conflict of Interest

The authors declare that the research was conducted in the absence of any commercial or financial relationships that could be construed as a potential conflict of interest.

## Publisher's Note

All claims expressed in this article are solely those of the authors and do not necessarily represent those of their affiliated organizations, or those of the publisher, the editors and the reviewers. Any product that may be evaluated in this article, or claim that may be made by its manufacturer, is not guaranteed or endorsed by the publisher.
