# Supplementary material for: Association Between the COVID-19 Pandemic and Infant Neurodevelopment: A Comparison Before and During COVID-19
Source: Front Pediatr. 2021 Oct 6;9:662165. doi: 10.3389/fped.2021.662165 (PMC8527007; doi:10.3389/fped.2021.662165)
Supplement: Supplementary file 1 [file Data_Sheet_1.docx]

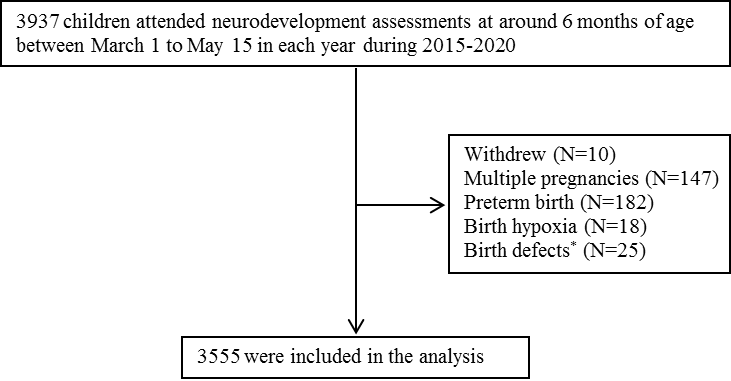


**(A)**


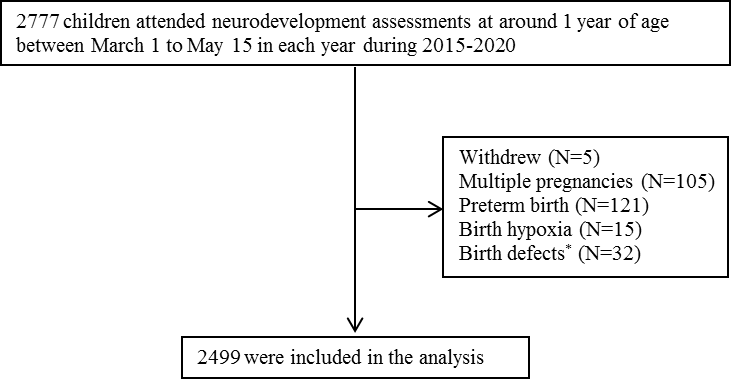


**(B)**

**Supplementary Figure 1.** Selection process of the present study. A: children attending neurodevelopment assessments at around six months of age; B: children attending neurodevelopment assessments at around one year of age. ^*^Restricted to birth defects that might affect neurodevelopmental outcomes (including major birth defects and anomalies that affect language and motor functions).

**Supplementary Table 1.** The prevalence (%) of neurodevelopmental delay by age, year, and birth order.

| **Cognitive at 6 months** | | | | **Cognitive at 1 year** | | | |
| --- | --- | --- | --- | --- | --- | --- | --- |
| Year | All | First-born | Later-born | Year | All | First-born | Later-born |
| 2015 | 6·6 | 6·9 | 4·3 | 2015 | 3·1 | 3·0 | 5·0 |
| 2016 | 4·4 | 3·2 | 9·5 | 2016 | 9·4 | 10·2 | 5·6 |
| 2017 | 2·4 | 2·4 | 2·3 | 2017 | 1·6 | 1·4 | 2·2 |
| 2018 | 4·9 | 4·6 | 5·2 | 2018 | 4·7 | 5·6 | 3·5 |
| 2019 | 2·6 | 2·1 | 3·5 | 2019 | 2·9 | 2·5 | 3·7 |
| 2020 | 3·5 | 3·1 | 4·4 | 2020 | 5·6 | 5·7 | 5·4 |
| Total | 4·0 | 3·8 | 4·4 | Total | 4·5 | 4·8 | 3·9 |
| **Gross motor at 6 months** | | | | **Gross motor at 1 year** | | | |
| Year | All | First-born | Later-born | Year | All | First-born | Later-born |
| 2015 | 7·9 | 7·8 | 8·5 | 2015 | 15·4 | 16·1 | 5·0 |
| 2016 | 11·9 | 12·1 | 10·8 | 2016 | 20·8 | 21·3 | 18·5 |
| 2017 | 4·9 | 4·8 | 5·1 | 2017 | 16·3 | 17·3 | 13·3 |
| 2018 | 8·1 | 6·4 | 9·9 | 2018 | 16·5 | 16·2 | 16·9 |
| 2019 | 5·7 | 4·9 | 7·0 | 2019 | 14·2 | 14·0 | 14·5 |
| 2020 | 9·2 | 7·3 | 13·7 | 2020 | 13·3 | 14·8 | 9·3 |
| Total | 7·7 | 7·1 | 8·9 | Total | 15·9 | 16·3 | 15·0 |
| **Fine motor at 6 months** | | | | **Fine motor at 1 year** | | | |
| Year | All | First-born | Later-born | Year | All | First-born | Later-born |
| 2015 | 11·3 | 11·6 | 8·5 | 2015 | 2·5 | 2·3 | 5·0 |
| 2016 | 10·9 | 10·2 | 13·5 | 2016 | 6·2 | 5·5 | 9·3 |
| 2017 | 4·8 | 4·3 | 5·6 | 2017 | 2·2 | 2·9 | 0·0 |
| 2018 | 9·2 | 8·3 | 10·4 | 2018 | 1·6 | 2·1 | 1·0 |
| 2019 | 6·2 | 6·4 | 5·8 | 2019 | 0·8 | 0·3 | 1·6 |
| 2020 | 7·3 | 6·8 | 8·7 | 2020 | 6·0 | 4·8 | 9·3 |
| Total | 8·1 | 7·9 | 8·4 | Total | 2·6 | 2·6 | 2·7 |
| **Communication at 6 months** | | | | **Communication at 1 year** | | | |
| Year | All | First-born | Later-born | Year | All | First-born | Later-born |
| 2015 | 1·1 | 1·0 | 2·1 | 2015 | 27·9 | 28·4 | 20·0 |
| 2016 | 0·3 | 0·3 | 0·0 | 2016 | 33·2 | 32·0 | 38·9 |
| 2017 | 0·5 | 0·5 | 0·5 | 2017 | 18·5 | 15·8 | 26·7 |
| 2018 | 0·7 | 0·6 | 0·8 | 2018 | 26·5 | 26·8 | 26·0 |
| 2019 | 1·2 | 1·9 | 0·0 | 2019 | 25·5 | 24·1 | 27·7 |
| 2020 | 1·5 | 1·0 | 2·5 | 2020 | 35·8 | 34·3 | 40·0 |
| Total | 0·9 | 0·9 | 0·8 | Total | 27·7 | 27·2 | 28·7 |
| **Personal-social at 6 months** | | | | **Personal-social at 1 year** | | | |
| Year | All | First-born | Later-born | Year | All | First-born | Later-born |
| 2015 | 11·9 | 11·9 | 12·8 | 2015 | 7·2 | 7·4 | 5·0 |
| 2016 | 16·8 | 17·3 | 14·9 | 2016 | 6·5 | 7·1 | 3·7 |
| 2017 | 11·2 | 10·7 | 12·2 | 2017 | 7·1 | 7·9 | 4·6 |
| 2018 | 9·0 | 7·7 | 10·7 | 2018 | 4·2 | 3·7 | 4·8 |
| 2019 | 8·9 | 8·5 | 9·7 | 2019 | 1·8 | 1·2 | 2·8 |
| 2020 | 9·7 | 9·1 | 11·2 | 2020 | 6·7 | 5·7 | 9·3 |
| Total | 10·7 | 10·5 | 11·2 | Total | 4·7 | 4·8 | 4·5 |
| **Poor neurodevelopmental outcome^a^ at 6 months** | | | | **Poor neurodevelopmental outcome^a^ at 1 year** | | | |
| Year | All | First-born | Later-born | Year | All | First-born | Later-born |
| 2015 | 5·6 | 5·5 | 6·4 | 2015 | 3·1 | 3·0 | 5·0 |
| 2016 | 5·2 | 4·5 | 8·1 | 2016 | 7·8 | 7·9 | 7·4 |
| 2017 | 1·5 | 1·9 | 0·9 | 2017 | 3·9 | 5·1 | 0·0 |
| 2018 | 3·2 | 2·4 | 4·2 | 2018 | 2·7 | 2·3 | 3·2 |
| 2019 | 2·2 | 2·1 | 2·3 | 2019 | 1·4 | 1·0 | 2·0 |
| 2020 | 2·4 | 1·6 | 4·4 | 2020 | 3·9 | 2·9 | 6·8 |
| Total | 3·1 | 2·9 | 3·5 | Total | 3·3 | 3·2 | 3·3 |

^a^Defined as developmental delay in more than two domains.
